# Burden of Respiratory Syncytial Virus (RSV) Infection Among Adults in Nursing and Care Homes: A Systematic Review

**DOI:** 10.1111/irv.70008

**Published:** 2024-09-16

**Authors:** Richard Osei‐Yeboah, Stephen Amankwah, Elizabeth Begier, Miranda Adedze, Franklin Nyanzu, Pious Appiah, Jochebed Ode Boakye Ansah, Harry Campbell, Reiko Sato, Luis Jodar, Bradford D. Gessner, Harish Nair

**Affiliations:** ^1^ Centre for Global Health, Usher Institute University of Edinburgh Edinburgh UK; ^2^ Institute of Biochemistry and Biophysics Polish Academy of Sciences Warsaw Poland; ^3^ Global Medical Development Scientific and Clinical Affairs Pfizer Vaccines Dublin Ireland; ^4^ Sahlgrenska Academy University of Gothenburg Gothenburg Sweden; ^5^ Department of Epidemiology and Disease Control, School of Public Health University of Ghana Accra Ghana; ^6^ Department of Medical Microbiology, Medical School, College of Health Sciences University of Ghana Accra Ghana; ^7^ Department of Population Health University of Toledo Toledo Ohio USA; ^8^ Value & Evidence Pfizer Inc Collegeville Pennsylvania USA; ^9^ Vaccines Medical Development, Scientific and Clinical Affairs Pfizer Inc Collegeville Pennsylvania USA; ^10^ MRC/Wits Rural Public Health and Health Transitions Research Unit (Agincourt), School of Public Health, Faculty of Health Sciences University of the Witwatersrand Johannesburg South Africa; ^11^ School of Public Health Nanjing Medical University Nanjing People's Republic of China

**Keywords:** adults, and care home, burden, infection, nursing, respiratory syncytial virus

## Abstract

**Background:**

Older adults in nursing and care homes (NCHs) are vulnerable to severe respiratory syncytial virus (RSV) infection, hospitalization, and death. This study aimed to gather data on RSV disease among older adults in NCHs and identify reported risk factors for RSV hospitalization and case fatality.

**Methods:**

The study protocol was registered in PROSPERO (CRD42022371908). We searched MEDLINE, EMBASE, and Global Health databases to identify articles published between 2000 and 2023. Observational and experimental studies conducted among older adults in NCHs requiring assistive care and reporting RSV illness were included and relevant data were extracted.

**Results:**

Of 18,690 studies screened, 32 were selected for full‐text review, and 20 were included. Overall, the number of NCH residents ranged from 42 to 1459 with a mean age between 67.6 and 85 years. Attack rates ranged from 6.7% to 47.6% and annual incidence ranged from 0.5% to 14%. Case fatality rates ranged from 7.7% to 23.1%. We found similar annual incidence rates of RSV‐positive acute respiratory infection (ARI) of 4582 (95% CI: 3259–6264) and 4785 (95% CI: 2258–10,141) per 100,000 reported in two studies. Annual incidence rate of RSV‐positive lower respiratory tract infection was 3040 (95% CI: 1986–4454) cases per 100,000 adults. Annual RSV‐ARI hospital admission rates were between 600 (95% CI: 190–10,000) and 1104 (95% CI: 350–1930) per 100,000 person‐years. Among all RSV disease cases, commonly reported chronic medical conditions included chronic obstructive pulmonary disease (COPD), heart failure, ischemic heart disease, coronary artery disease, hypertension, diabetes, kidney dysfunction, cerebrovascular accident, malignancies, dementia, and those with a Charlson comorbidity score > 6.5.

**Conclusion:**

Data on RSV infection among NCH residents are limited and largely heterogeneous but document a high risk of illness, frequent hospitalization, and high mortality. Preventive interventions, such as vaccination, should be considered for this high‐risk population. Nationally representative epidemiologic studies and NCH‐based viral pathogen surveillance could more precisely assess the burden on NCH residents.

## 1 Introduction

Respiratory syncytial virus (RSV) is a major cause of respiratory tract infections in infants, older adults, and other high‐risk groups. A recently published meta‐analysis from the United States reported a pooled RSV‐related hospitalization incidence from prospective studies of 282 per 100,000 in adults aged 65 years and above [1] after adjustment for reduced sensitivity of polymerase chain reaction (PCR) nasopharyngeal/nasal swab testing, which is an issue among older adults [2, 3]. Previous studies suggest that RSV‐attributable morbidity and mortality among older adults may be similar to seasonal influenza epidemics [4, 5]. Large‐scale epidemiological studies on the impact of RSV and influenza virus on hospitalization and mortality rates in older adults reported findings similar to the previous studies [6, 7, 8]. Novel estimates of RSV‐associated hospital admission in adults in the European Union show that about 92% of admissions are reported in adults older than 65 years [9]. Further, a recent global meta‐analysis projected that 787,000 hospitalizations in adults aged 65 years and above are occurring annually in high‐income countries alone [10]. Older adults with chronic respiratory diseases, heart failure, and immunosuppressing conditions are at higher risk from severe or fatal RSV infections [11, 12, 13]. Immunosenescence and decreased levels of RSV‐specific neutralizing antibodies in the serum also contribute to higher susceptibility to severe RSV infections [14, 15, 16, 17].

Nursing and care home (NCH) residents may have an increased risk of severe RSV infections, compared with community‐dwelling persons of equivalent age [11]. This may be due to the increased prevalence of comorbidities in NCH residents, which is associated with severe RSV infection, including neuropsychiatric, cardiovascular, gastrointestinal disorders, hypertension, vascular disease, arthritis, depression, and gastroesophageal reflux disease [12, 13]. It may also be due to increased frailty [4], and increased risk of acquiring RSV infection in a closed environment.

Recent reviews have focused predominantly on epidemiological, clinical, diagnostic, and therapeutic data and prevention options for RSV infection among older adults [5, 6, 7] but only a few existing reviews have focused on RSV burden among NCH residents. We aimed to gather data on RSV infections among older adults in NCHs, report the burden of disease including incidence rates and associated case fatality rates, and identify reported risk factors for hospitalization. A better understanding of RSV burden in NCHs will be critical to prioritize preventive vaccination campaigns and ensure that access to novel antivirals is readily available to treat this high‐risk populations.

### 1.1 Study Objectives and Registration

The study was conducted in line with the Preferred Reporting Items for Systematic Reviews and Meta‐Analyses (PRISMA) guidelines [8]. The study was registered in PROSPERO with reference number CRD42022371908. This study did not require institutional review board approval.

### 1.2 Data Searches

We conducted a systematic literature review to identify up‐to‐date data published on RSV infections in NCHs across the globe using pre‐defined search queries. We systematically searched three databases—Ovid MEDLINE, Ovid EMBASE and Ovid Global Health (1973 onwards) for original studies published on RSV infections in NCHs from January 1, 2000, to June 30, 2023. We hand‐searched the reference lists of all included studies and other relevant studies to identify additional eligible studies. We did not apply language restrictions to our searches. The queries used for the database searches are included in the supporting information.

### 1.3 Study Screening and Selection

#### 1.3.1 Inclusion Criteria

We included papers reporting RSV disease occurrence, prevalence, or incidence rates among residents of nursing, care, residential, or retirement homes where residents require assistive care. We considered papers using experimental–randomized controlled trials (RCTs) and observational–cohort, cross‐sectional, case–control study designs. We included studies that confirmed RSV using diagnostic tests such as PCR, cell culture, immunofluorescence, and other rapid antigen‐based tests. Studies reporting RSV infection frequency from RSV serology (e.g., pre‐and‐post‐RSV season serology or acute and convalescent specimens) and studies reporting 28‐day post‐RSV infection mortality were considered.

#### 1.3.2 Exclusion Criteria

We excluded studies that did not report on RSV disease in NCHs. We excluded case series and case reports describing clinical presentations attributable to RSV, studies reporting RSV infections only among adults with chronic medical conditions, studies only reporting geriatric medical facility or hospital‐acquired infections, reviews or systematic reviews or scoping reviews (references were hand‐searched), and proceedings from conferences (unless in a peer‐reviewed publication as a full research paper).

### 1.4 Title and Abstract Screening

At least two reviewers (ROY, SA, MA, FN, PA, and JOBA) screened the titles and abstracts of each study using the Covidence App (©2023). Conflicts resulting from the inclusion and exclusion of studies were resolved through discussion and consensus. Eligible studies were selected for full‐text review.

### 1.5 Full‐Text Review

At least two reviewers (ROY, SA, MA, FN, PA, and JOBA) screened the full‐text of each selected study for eligibility using the Covidence App (2023). Studies meeting the inclusion criteria were selected for data extraction. Any conflicts regarding inclusion of studies or extracted data were resolved through discussion and consensus. We hand‐searched the reference lists of included papers to identify any relevant missing papers.

### 1.6 Risk of Bias Assessments and Data Extraction

For each of the selected studies, two reviewers (ROY and SA) independently assessed for risk of bias using the Cochrane Risk of Bias 2 (RoB 2) tool for RCTs [14] and the Risk of Bias in Non‐Randomized studies of Exposure (ROBINS‐E) tool for observational studies [15]. Risk of bias plot for RoB 2 was generated using the *robvis* package in RStudio (version 1.2.5019) on R Software (version 4.1.2) [16]. We extracted relevant data from the selected studies that included the study characteristics, outcome measures, the RSV subtypes where available, risk factors, and control measures. We calculated the RSV incidence/attack rates (IR/AR) from studies that did not directly report these data where necessary.

### 1.7 Data Analysis

We conducted a descriptive analysis of the data (number and age of NCH residents, date, and location of study), diagnostic tests involved, and the timeframe of RSV assessment (seasonal only, entire year). We could not perform a meta‐analysis due to the high level of heterogeneity in the data between countries, differences in outcome measure reporting, and the smaller number of cases reported in some studies.

## 2 Results

**FIGURE 1:**
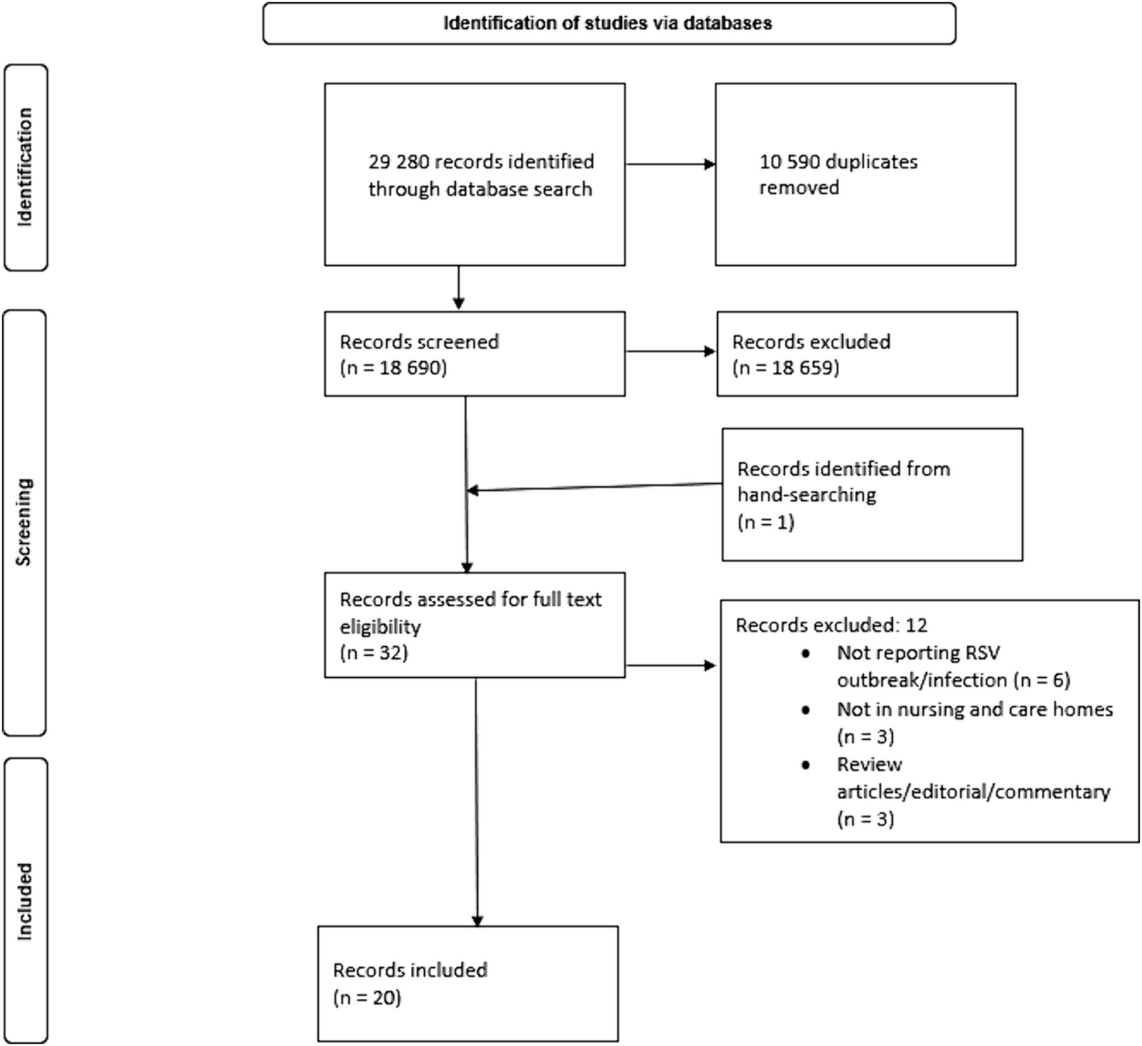
PRISMA flowchart outlining the search records of RSV disease in NCHs.

We screened the titles and abstracts of 18,690 studies of which 31 were eligible for full‐text assessment. We identified an additional article for full‐text screening from a hand search of reference lists of the selected studies. Of the 32 articles, 20 were eligible for inclusion in the study (Figure 1). Studies were published between April 18, 2000, and March 1, 2023. Of the 20 included studies, nine were conducted in the United States [17, 18, 19, 20, 21, 22, 23, 24, 25], two in Canada [26, 27], one each in Japan [28], Netherlands [29], England [30], Czech Republic [31], Portugal [32], Hong Kong [33], France [34], Slovenia [35], and one multi‐country (Belgium, Estonia, Germany, Spain, United Kingdom, and United States) [11].

### 2.1 Overview of Study Characteristics

The total number of nursing home residents studied in the 20 included studies combined was 10,114 (range 42 to 1459, mean 423) from over 694 NCHs. The mean age of the study participants was between 67.6 and 85 years and median age between 73 and 88 years. There were seven studies reporting RSV disease attack rates [17, 18, 19, 26, 28, 29, 30] and 13 studies reporting RSV incidence rates, incidence proportion, or prevalence estimates [11, 20, 21, 22, 23, 24, 25, 27, 31, 32, 33, 34, 35].

### 2.2 RSV Disease Attack Rates in NCHs

Of the seven studies reporting RSV disease attack rates, there were three retrospective cohort, three prospective cohort and one mixed (prospective and retrospective) cohort study. The mean age of NCH residents in these studies ranged from 68.1 to 85 years. The number of NCH residents at risk of RSV infection ranged from 10 to 1459, and laboratory‐confirmed RSV cases ranged between 4 and 24. The overall RSV AR ranged from 6.7% to 47.6% (Table 1).

**TABLE 1:** Description of published studies reporting RSV disease occurrence in NCHs.

Source (author/year)	Country	Study design	Study period	Age, mean (SD)/median (IQR)	No. of residents	No. of infected	Incidence^a^/attack rate (IR/AR)/prevalence	Mode of viral confirmation
Najeros Pérez et al., 2023 [11]	Europe and United States	Prospective cohort	Season 1 (October 2019 to March 2020)	82.7 (8.5)	664	15	IR: 4785 cases per 100,000 person‐years (95% CI: 2258–10,141) AR: 2.26%	RT‐PCR
			Season 2 (October 2020 to June 2021)	83.4 (8.4)	494	1	IR: 291 cases per 100,000 person‐years (95% CI: 40–2097) AR: 0.20%	
Barrett et al., 2020 [17]	United States	Retrospective cohort	January 24 to February 24, 2019	73 (IQR: 47–89)	42	20	AR: 47.6%	Rapid RT‐PCR
Caram et al., 2009 [18]	United States	Prospective cohort	January 29 to February 26, 2008	68.1 (16.8)	52	7	AR: 13.5%	Culture, direct fluorescent antibody test, RT‐PCR
Spires et al., 2017 [19]	United States	Prospective cohort	January 5–21, 2015	Mean 81.3	41	6	AR: 42.9%^b^	RT‐PCR
Checovich et al., 2020 [20]	United States	Randomized control trial	2016–2017, 2017–2018, 2018–2019	83.7 (9.9)	497	38	Incidence: 7.6%	RT‐PCR
Ellis et al., 2003 [21]	United States	Retrospective cohort	August 1995 to July 1999	≥65	381 (Nursing homes) 81,885‐person years	1105	RSV‐positive tests: 1120 per week per 100,000 (in influenza season); 960 per week per 100,000 (in RSV season); 30 per week per 100,000 (in non‐winter‐viral season)	Culture, antigen‐positive test
Falsey et al., 2008 [22]	United States	Observational	1998–2000	85.3 (67–102)	452	25 out of 382 tested	Incidence: 6.5%^c^	Enzyme immunoassay
McElhaney et al., 2004 [23]	United States	Randomized control trial	Study 1 (February to March 2000)	Study 1: 81.3 (8.2)	Study 1: 89	Study 1: 0	Study 2 incidence: 2.8%	Culture
			Study 2 (December 2000 to March 2001)	Study 2:83.6 (6.5)	Study 2:109	Study 2:3		
O'Neil et al., 2019 [24]	United States	Prospective cohort	December 2, 2015, to April 30, 2016	67.6 (1.1)	105	2	Incidence: 2.0%	rRT‐PCR
Diaz‐Decaro et al., 2018 [25]	United States	Prospective cohort	May to July 2015	Range: 65–85	832	1	Prevalence: 5.5%^d^	Multiplex nested PCR
Loeb et al., 2000 [26]	Canada	Mixed prospective and retrospective cohort	July 1, 1993 to June 30, 1996	Mean 85	75	5	AR: 6.7%^f^	Immunofluorescence microscopy
Johnstone et al., 2014 [27]	Canada	Prospective cohort	September and October 2009, 2010, and 2011	86 (80–90)	1072	12 out of 87 symptomatic residents	Incidence: 14%^e^	Multiplex respiratory viral panel
Doi et al., 2014 [28]	Japan	Retrospective cohort	Mid‐January 2014	81.5 (8.5)	99	24	AR: 24%	RT‐PCR
Meijer et al., 2013 [29]	Netherlands	Prospective cohort	December 2012	Mean 84	10	4	AR: 38%	PCR
Yip et al., 2018 [30]	England	Retrospective observational	July 28, 2016, to March 27, 2017	Not specified	1459	13 RSV cases from 168 individual ILI outbreaks^g^	AR: 20.5% (95% CI: 15.4–23.1)^g^	Microbiological
Beran et al., 2021 [31]	Czech Republic	Prospective cohort	Season 1 (August 1, 2003, to May 31, 2004)	80.46 (7.04)	1249	0	^h^IR (ARI): 4582 cases (95% CI: 3259–6264) per 100,000 person‐years; IR (LRTI): 3040 cases (95% CI: 1986–4454) per 100,000 person‐years	PCR
			Season 2 (August 1, 2004, to May 20, 2005)	81.20 (6.61)	1277			
Chasqueira et al., 2018 [32]	Portugal	Prospective cohort	November 2013 to April 2014	Male: 83 (9.2) Female: 85 (7.2)	1022	5	Incidence: 0.5%	PCR
Hui et al., 2008 [33]	Hong Kong	Prospective cohort	April 2006 to March 2007	84.9 (8.9)	771	21	Incidence: 9.3%^i^	Rapid multiplex nested PCR and paired serology
Masse et al., 2017 [34]	France	Cross‐sectional	2013/2014–2016/2017 (December 1, 2013, to April 16, 2017)	88 (63–103)	107	3	Incidence: 2.8%	RT‐qPCR
Ursic et al., 2016 [35]	Slovenia	Prospective cohort	December 5, 2011, to May 31, 2012	84 (79.8–88.8)	90	5 out of 56 residents	Incidence: 8.9%^j^	PCR

^a^ Incidence rates are reported per person‐years, attack rates, incidence proportion, and prevalence in %.

^b^ RSV and/or human metapneumovirus (HMPV) were identified in 30 of the residents through epidemiologic investigation. Laboratory testing was done for 14 residents out of 30 and 6 were positive for RSV. The RSV attack rate was calculated from the laboratory‐confirmed cases [19].

^c^ A total of 617 residents were enrolled of which 452 completed the study and 382 had paired sera at baseline and 53 weeks for viral analysis [22].

^d^ Symptomatic ARIs in 52 residents; 10 of 52 residents had detectable viral pathogen; RSV was confirmed in one resident from one of three NCHs. The total period prevalence was stratified per NCH facility and target pathogen [25].

^e^ All RSV cases were reported in a single year [26].

^f^ A total of 269 nasopharyngeal swaps were obtained from 233 symptomatic residents. Nasopharyngeal swaps positive for respiratory viruses were 87 (symptomatic residents) of which 12 were RSV [27].

^g^ Total of 168 viral respiratory outbreaks of which RSV accounted for 13 cases. The median RSV cases per outbreak was 9 cases (IQR: 6–11). The overall number of individuals infected by RSV in the 13 outbreaks was not provided. The attack rate was reported for residents and excluded staff [30].

^h^ Incidence rate of RSV‐positive acute respiratory infection (ARI) and lower respiratory tract infection (LRTI) per 100,000 person‐years [31].

^i^ A total of 259 episodes of influenza‐like illnesses (ILI) in 194 residents. Nasopharyngeal aspirates were done in 256 out of 259 episodes. The total count of pathogens (bacteria and viruses) isolated throughout the episodes was 227 of which 21 RSV cases were confirmed [33].

j Of 90 residents there were 56 ARIs, 34 were confirmed for respiratory viruses and five RSV cases were identified [35].

### 2.3 RSV Disease Incidence Rates, Incidence Proportion, and Prevalence Estimates in NCHs

Of the 13 studies reporting incidence rates, incidence proportion, and prevalence estimates, there were two randomized controlled trials (RCTs) and 11 observational studies: nine prospective cohort studies, one retrospective cohort study, and one cross‐sectional study (Table 1). The study participants ranged from 90 to 1249 residents. The mean age was between 67.6 and 84.9 years whilst the median age was between 84 and 88 years.

The overall RSV disease incidence proportion ranged widely from 0.5 to 14%. Two studies reported RSV‐positive ARI which ranged between 4582 (95% CI: 3259–6264) per 100,000 person‐years [31] and 4785 (95% CI: 2258–10,141) per 100,000 person‐years [16]. RSV‐positive lower respiratory tract infections (LRTIs) episode incidence rate was 3040 (95% CI: 1986–4454) cases per 100, 000 person‐years [31].

Ellis et al., report that weekly RSV‐positive tests varied per season—1120 per week per 100,000 person‐years (in influenza season), 960 per week per 100,000 person‐years (in RSV season), and 30 per week per 100,000 person‐years (in a non‐winter season) [21]. In this study, RSV season excluded dates classified as influenza season and in each of the seasons, the dates of influenza season were completely overlapped by RSV season, which would cause substantial underestimation of RSV cases.

### 2.4 RSV‐Associated Case Fatality

Overall case fatality rate ranged from 7.7% to 23.1% [18, 19, 30, 31, 32, 33] (Table 2). Only one study reported population‐based mortality rates [21]. In this study, the reported mortality rate among non‐high‐risk groups was 1630 (95% CI: 1070–2200) per 100,000 person‐years, whilst high‐risk groups had a mortality rate of 1730 (95% CI: 1150–2330) per 100,000 person‐years [21] (Table 2). High‐risk groups were defined as all persons and events following a medical encounter with diagnostic or procedure codes indicating asthma/chronic lung disease, diabetes mellitus, chronic heart disease, cancer/immunocompromised, human immunodeficiency virus, and chronic renal or liver disease or filled prescriptions for the treatment of these conditions. Non–high‐risk groups included all other persons and events [21].

**TABLE 2:** Case definition, RSV‐associated hospitalization, length of stay, and case fatality.

Source (author/year)	Case definition	No. of hospitalization (%)	Length of stay (days)	No. of fatality (%)
Najeros Pérez et al., 2023 [11]	An ARI was defined as a respiratory infection when at least 2 of the following signs and/or symptoms occurred together: rhinorrhea/nasal congestion, sore throat, cough (new or increasing), sputum production (new or increasing), shortness of breath or dyspnea (new or increasing), wheezing (new or increasing), or feverishness or fever (temperature ≥37.5°C). Confirmed RSV‐ARI was defined as an ARI episode with detection of RSV by RT‐PCR in a combined nasal and throat swab.	0	0	0
Barrett et al., 2020 [17]	Community living center resident who developed upper respiratory symptoms (rhinorrhea and cough) and tested positive for RSV.	4 (20%)	1–9 days	0 (0%)
Caram et al., 2009 [18]	Three respiratory symptoms (new‐onset or increase in chronic cough, new‐onset or increase in sputum, dyspnea, chills, headache, myalgias, malaise, sore throat, or nasal congestion) or two respiratory symptoms and temperature of 38.01°C or greater.	Not specified	Not specified	1 (14.3%)
Spires et al., 2017 [19]	Symptoms: Fever ≥37.8°C (100.0°F), cough, dyspnea, rhinorrhea or sneezing, hoarseness, congestion, fatigue, and malaise.	Not specified	Not specified	5 (16.7%)
Checovich et al., 2020 [20]	Characterized by any 2 of the following symptoms: cough, nasal congestion, rhinorrhea/runny nose, sore throat, or fever.	Not specified	Not specified	Not specified
Ellis et al., 2003 [21]	Not defined	No high risk: 600 (190–1000) per 100,000 person‐years; High‐risk: 1140 (350–1930) per 100,000 person‐years	Not specified	No high risk: 1603 (1070‐2200) per 100,000 person‐years; High risk: 1730 (1150–2330) per 100,000 person‐years
Falsey et al., 2008 [22]	Respiratory tract infections were categorized according to standard definitions and included common cold, influenza‐like illness, pharyngitis, otitis media, sinusitis (defined as upper respiratory infection (URI)) and acute bronchitis and pneumonia (defined as LRI).	Not specified	Not specified	Not specified
McElhaney et al., 2004 [23]	The new onset of two symptoms: one respiratory symptom (cough, sore throat, nasal or sinus congestion, or runny nose) and one additional respiratory symptom or one constitutional symptom (feverishness, chills/sweats, myalgia, fatigue, headache, poor endurance, or increased shortness of breath).	Not specified	Not specified	Not specified
O'Neil et al., 2019 [24]	Fever ≥37.3°C (99.1°F), headache, sore throat, shortness of breath, chills, muscle and/or joint pain, coughing, wheezing, fatigue, congestion, or runny nose, or change of mental status or confusion.	Not specified	Not specified	Not specified
Diaz‐Decaro et al., 2018 [25]	Clinical symptoms suggestive of influenza‐like illness (ILI), that is, fever, congestion, rhinorrhea, cough (with or without sputum production), shortness of breath, or other pulmonary complaints (pleurisy and wheezing)	Not specified	Mean: 60 days^a^	Not specified
Loeb et al., 2000 [26]	Upper respiratory tract infection was defined by the presence of at least 2 of the following signs or symptoms: runny nose or sneezing; nasal congestion; sore throat, hoarseness, or difficulty swallowing; dry cough; and cervical lymphadenopathy. Lower respiratory tract infection was defined by the presence of at least 3 of the following: new or increased cough; new or increased sputum production; fever (temperature >38°C); pleuritic chest pain; new or increased findings on chest examination; and one of the following: new or increased shortness of breath, a respiratory rate of more than 25 breaths/min, or worsening mental or functional status. Pneumonia was defined by the presence of compatible radiological findings and at least 2 of the above symptoms or signs.	Not specified	Not specified	0 (0%)
Johnstone et al., 2014 [27]	Fever (≥38 C), worsening cough, nasal congestion, sore throat, headache, sinus problems, muscle aches, fatigue, ear ache or infection, chills, not otherwise explained by an alternative diagnosis	Not specified	Not specified	Not specified
Doi et al., 2014 [28]	Not defined	Not specified	Not specified	Not specified
Meijer et al., 2013 [29]	Not defined	Not specified	Not specified	Not specified
Yip et al., 2018 [30]	According to Public Health England guideline	3 care homes reporting admissions	Not specified	3 (23.1%) care homes
Berran et al., 2021 [31]	ARI: Patients requiring medical attention and presenting ≥ 4 of the following signs/symptoms: nasal congestion, sore throat, cough, sputum, dyspnea, rhinorrhea, wheezing, rales, rhonchi, and fever (axillary temperature ≥38.0°C in season 1 and ≥37.5°C in season 2); LRTI: Diagnosis of bronchitis, broncho‐pneumonia or pneumonia confirmed by X‐ray.	4 (10.3%)	Median: 18.5 (14.0–37.0)	3 (7.7%)
Chasqueira et al., 2018 [32]	Sudden onset of symptoms, at least one respiratory symptom (cough, sore throat, shortness of breath, and coryza), and a clinician's judgment that the illness was due to an infection.	Not specified	Not specified	1(20%)
Hui et al., 2008 [33]	Clinical presentation consisting of fever >37.8°C or an acute deterioration in physical or mental ability, plus either new onset of one or more respiratory symptoms or an acute worsening of a chronic condition involving respiratory symptoms.	Not specified	Not specified	3 (12%)
Masse et al., 2017 [34]	Person developing sudden onset of any constitutional symptom, in addition to any respiratory sign.	Not specified	Not specified	Not specified
Ursic et al., 2016 [35]	Upper respiratory tract infection (URTI) or lower respiratory tract infection (LRTI).	Not specified	Not specified	Not specified

^a^ Relates to all residents who tested for respiratory tract infection and are not specific to RSV.

### 2.5 RSV Subtype

Five of 20 studies reported frequency of RSV subtypes (i.e., RSV A/B). RSV‐A alone infection was identified in one case [11]. Both RSV‐A and RSV‐B infections were reported in two studies—RSV‐A was identified in the majority of the cases: 28/39 cases (71.8%) and in 10/12 cases (83.3%) [27]. For the other two studies, RSV‐B alone was identified in 4/4 cases (100%) [29], and in 6/14 (42.9%) laboratory‐tested cases [19].

### 2.6 Clinical Characteristics and Chronic Medical Conditions Among RSV Cases

The medical history and clinical characteristics of RSV disease case‐patients included current or a history of smoking, mobility limitations (bed or wheelchair‐bound), clinical characteristics (fever [37.7°C–40.0°C], wheezing, rhinorrhea, sore throat, malaise, change in sputum production, new or increased cough, myalgia, hoarseness, and sneezing), pleuritic chest, and pneumonia [17, 18, 26, 28, 29]. Tube feeding [33] and polypharmacy (five medications or more) [27] were other patient characteristics described among individuals with RSV‐positive tests. Common chronic medical conditions reported among RSV cases compared with non‐cases were (congestive) heart failure, COPD, respiratory allergy, hypertension, diabetes mellitus, kidney dysfunction, malignancy, dementia, cerebrovascular accident, ischemic heart disease, coronary artery disease, end‐stage renal disease, and multiple comorbidities resulting in a Charlson comorbidity score greater than 6.5.

### 2.7 Risk Factors for RSV Infection

One study [31] reported risk factors for RSV infection and these included being a female, odds ratio (OR): 4.98 (95% confidence interval [CI]: 1.62–15.33); chronic heart failure, OR: 2.31 (1.13–4.73), and diabetes requiring insulin, OR: 9.82 (2.20–43.90).

### 2.8 RSV Hospitalization and Length of Stay

Two studies [17, 30] reported RSV cases that required hospitalization (Table 2). Four out of 20 (20%) and 3/13 (23.1%) RSV cases required hospitalization, respectively [17, 30]. One study reported that 4/39 RSV cases (10.3%) required hospitalization [31] (Table 2). Among residents classified as not high risk, the RSV hospitalization rate was 600 (95% CI: 190–1000) per 100,000 person‐years and the hospitalization rate among residents classified as high‐risk was 1140 (95% CI: 350–1930) per 100,000 person‐years [21].

The overall length of stay in the hospital was 1 to 9 days [17] and a median of 18.5 (IQR: 14–37 days) [31] (Table 2). One study reported a mean length of stay of 60 days, but this related to all residents testing positive for any viral respiratory tract infection and not specific to RSV [25].

### 2.9 Diagnostic Tests

The diagnostic tests for RSV included a combination of rapid real‐time PCR, and reverse transcription PCR (RT‐PCR), as well as other low‐sensitivity testing modalities such as viral culture, direct fluorescent antibody (DFA) test, and immunofluorescence microscopy (IFM). Two studies used either only IFM or relied on microbiological evidence of pathogens such as culture [26, 30]. In some settings, RSV and other viral pathogens were confirmed by either only or a combination of PCR (including reverse transcription quantitative PCR [RT‐qPCR]), viral culture, antigen‐positive tests, enzyme immunoassay, rapid multiplex nested PCR, and paired serology.

### 2.10 Management and Control Measures

NCHs followed specific management and control protocols and the measures deployed across the NCHs were similar in some instances. Management and control measures included the relocation/cohorting of positive patients into single or double occupancy rooms, halting new admissions and group activities, daily environmental rounds, and high audits for hand hygiene [17]. Isolation and cohorting of infected and directly exposed residents involved the creation of distance between separate units [29]. Moving each symptomatic patient to a private room or to a room housing another case, all cases were placed on contact and droplet precautions [19]. Affected units were closed to new admissions and transfers [30]. Loeb et al. reported that treatment and transfer decisions for individual cases were at the discretion of the resident physician. Infection control measures were implemented during events considered as outbreaks according to the policies and procedures of each institution and the local public health departments [26]. One study reported that no control measure was deployed and no isolation or restriction of positive cases occurred [18]. The use of intravenous antibiotics, intravenous fluids, oxygen supplementation, and extensive involvement of allied health staff were used in managing RSV disease patients [33].

## 3 Discussion

We systematically reviewed studies regarding RSV infection among older adults in NCHs. Our study included 20 studies and present the most comprehensive assessment of the burden of RSV infection in NCHs completed to date, with 13 additional studies compared with a 2019 review [36]. While available data on the frequency and associated morbidity and mortality of RSV infection among NCH residents are largely heterogeneous and limited, including in some cases by use of low sensitivity testing modalities, it is nevertheless clear that NCH residents experience a high risk of illness, frequent hospitalization, and high mortality, indicating that preventive interventions, such as RSV vaccination, should be considered for this high‐risk population.

Whilst we found high variability of ARs in the studies included in this review, most studies showed that NCH residents have substantially higher RSV ARs than what has been reported in older adults. It is reported from previous studies that RSV ARs in NCHs could be 5%–10% per year with significant rates of pneumonia and deaths [37]. ARs in the studies included in this review were higher than annual incidence rates but both were at least several fold higher than published rates among community dwelling older adults. ARs reached 47.6%, which is about 5.3–6.6 times higher than ARs of 8.9% [38] and 4.2%–7.2% [39] reported in previous prospective cohorts of community‐dwelling older adults. A previous systematic review of burden of RSV among older adults in long‐term care including seven studies covering 1984 to 2016 found that RSV incidence proportions ranged from 1.1% to 13.5% [36]. The annual RSV disease incidence proportion in our review reached 14%, which is about twice the reported incidence in community‐dwelling older adults [39]. Seasonal RSV infection rate in a NCH was found to be higher in the influenza season (1120 cases per week per 100,000) compared with the RSV season (960 cases per week per 100,000) [21]; however, we observed inconsistencies in the reporting of cases where influenza and RSV seasons overlapped. RSV season excluded dates that overlapped with influenza season implying that more RSV cases were assigned to influenza season; therefore, the reported higher RSV infection rate in the influenza season may not be accurate in this population [40]. Alternatively, this may be driven by viral pathogen surveillance being predominately focused on influenza and thus testing is often increased during this time period.

Additionally, higher proportions of adults in long‐term care facilities than community‐dwelling adults were reported to be living with current chronic medical conditions—COPD, diabetes, vascular disorders, cardiac disorders, renal and urinary disorders, neoplasm, and respiratory, thoracic and mediastinal disorders [11]. Our systematic review documents that these chronic medical conditions are commonly reported among RSV cases compared with non‐RSV cases in NCHs.

Few studies reported RSV‐associated hospital admissions among NCH residents; among those that did, we found high rates of hospital admission and overall length of stay. The rates of hospital admission found among non‐high‐risk and high‐risk residents in our study (600 vs. 1140 per 100,000) were higher than rates reported from a pooled estimate of 347 (203–595) per 100,000 adults aged ≥65 years in high‐income countries after adjusting for under‐ascertainment [10]. Among the hospitalized RSV‐ARI cases, we found longer hospital lengths of stay in NCH residents (median: 18.5 days) compared with community‐dwelling older adults hospitalized with RSV (median: 9 days) [41]. Case‐fatality rates in NCHs were as high as 23.1%. Pooled RSV in‐hospital CFR estimates among community dwelling older adults in high‐income countries were 6.1% and 7.1% [10, 42], or 1/3 to 1/4 the CFR of non‐hospitalized NCH residents.

Similar to disease occurrence, this greatly increased risk of death in NCH residents likely reflects the underlying health of older adults living in NCHs, including greater comorbidities and advanced age. NCH residents are generally frail with reduced or impaired physical activity [43, 44] and may experience higher multimorbidity, increased disability, and disease complexity compared with the general population [4, 13, 43]. Reilev et al. report that most NCH residents in Norway had more than one chronic condition. In this study, the number of comorbidities a resident had was associated with low quality of life (QoL) scores, and those with multiple comorbidity (≥5 conditions) showed the greatest impact on their well‐being [45]. Other contributing factors may exist such as greater intensity of exposure in a relatively confined setting of NCHs.

Our study further identified risk factors—being a female, chronic heart failure and, diabetes requiring insulin—for RSV infection in this population. Similar risk factors for RSV‐associated hospital admissions and severe outcomes, especially underlying cardiopulmonary and metabolic conditions have been reported among community‐dwelling [46, 47].

The burden of RSV may be largely underestimated in older adults due to low levels of testing or diagnostic limitations such as viral culture and antigen detection insensitivity due to low viral titers [3, 37, 40], which like the results in reported rates being half of the true incidence even when comprehensive nasal/nasopharyngeal swab testing is conducted [2, 10]. In frail NCH residents, testing may also be limited for several reasons including the inability to obtain clinical specimens, which may potentially lead to an underestimation of the RSV burden. Further, there are multiple additional potential sources of underestimation of the incidence of RSV disease, such as the timing of surveillance and case definition used [40, 48]. Testing methods in the included studies were likely low sensitivity, which would imply that ARs and IRs were substantially greater than reported.

The risk of bias assessments of the included studies showed that the RCTs had an overall low risk of bias. However, there were some concerns about missing outcome data and the selection of the reported results, and a high risk of bias related to the measurement of outcomes (RSV infection/case positivity) (Figure S1). RCTs primarily focused on diagnostic tool/testing efficiency. For the observational studies, two studies showed an overall high risk of bias, four studies had some concerns overall and 12 studies showed a low risk of bias (Table S1). Most of the observational studies had concerns about the measurement of the outcome. We found that some studies did not clearly report missing data and the selection of the reported results in some studies was confusing. For instance, when RSV and influenza seasons overlapped, RSV cases were assigned to the influenza season. In one study, this led to higher counts of RSV cases in the influenza season than in the RSV season. Although we observed some biases in a few of the included studies, our findings highlight the substantial burden of RSV in NCHs in a high‐risk population and underscore the need to ensure the prevention of RSV infections and outbreaks in NCHs.

It is important to prioritize this population for RSV prevention and vaccination programs and priority setting. Two RSV vaccines, RSVPreF3‐AS01E and RSVpreF, have been approved for use in older adults aged over 60 years by the Food and Drugs Administration (FDA) in the United States and the European Medicines Agency (EMA) [49, 50, 51]. Post‐marketing surveillance to assess the likely impact of the introduction of RSV immunization on preventing severe infections and hospitalizations in this population will be necessary in the next few years.

## 4 Conclusion

Data on RSV infection among NCH residents are limited and largely heterogeneous but document a high risk of illness, frequent hospitalization, and high mortality. Preventive interventions, such as RSV vaccination, should be considered for this high‐risk population. Nationally representative epidemiologic studies and NCH‐based viral pathogen surveillance could more precisely assess the burden in NCH residents.

## Author Contributions

**Richard Osei‐Yeboah:** conceptualization, writing – original draft, methodology, formal analysis, investigation, data curation, visualization, validation, software. **Stephen Amankwah:** methodology, formal analysis, data curation, investigation, validation. **Elizabeth Begier:** funding acquisition, supervision, validation, project administration. **Miranda Adedze:** investigation, methodology, data curation. **Franklin Nyanzu:** investigation, methodology, data curation. **Pious Appiah:** investigation, methodology, data curation. **Jochebed Ode Boakye Ansah:** investigation, data curation, methodology. **Harry Campbell:** conceptualization, investigation, supervision, validation, project administration, methodology, resources. **Reiko Sato:** funding acquisition, supervision, project administration, methodology, resources. **Luis Jodar:** funding acquisition, validation, supervision, project administration, methodology, resources. **Bradford D. Gessner:** funding acquisition, validation, supervision, project administration, methodology, resources. **Harish Nair:** conceptualization, investigation, validation, supervision, project administration, methodology, resources.

## Conflicts of Interest

The authors declare no conflicts of interest.

### Peer Review

The peer review history for this article is available at https://www.webofscience.com/api/gateway/wos/peer‐review/10.1111/irv.70008.

## Supporting information


**Figure S1:** Risk of bias assessments of randomised controlled trials using the Cochrane Risk of Bias 2 (RoB 2) tool.
**Table S1:** Risk of bias in non‐randomised studies of exposure (ROBINS‐E) assessment for observational studies.

## Data Availability

All the data used in this review are presented.
