# Supplementary material for: Burden of Respiratory Syncytial Virus (RSV) Infection Among Adults in Nursing and Care Homes: A Systematic Review
Source: Influenza Other Respir Viruses. 2024 Sep 16;18(9):e70008. doi: 10.1111/irv.70008 (PMC11405123; doi:10.1111/irv.70008)
Supplement: Supplementary file 1 — Figure S1: Risk of bias assessments of randomised controlled trials using the Cochrane Risk of Bias 2 (RoB 2) tool. Table S1: Risk of bias in non‐randomised studies of exposure (ROBINS‐E) assessment for observational studies. [file IRV-18-e70008-s001.docx]

**Osei-Yeboah et al., 2024**

**Title: Burden of Respiratory syncytial virus (RSV) infection among adults in nursing and care homes: a systematic review**

**Supplementary file**

**Search terms:**

**Medline:**

1. *Respiratory Syncytial Viruses/ or *Respiratory Syncytial Virus Infections/ or respiratory syncytial.mp. or *Respiratory Syncytial Virus, Human/
2. infection.mp. or exp Infections/
3. outbreak.mp. or exp Disease Outbreaks/
4. incidence.mp. or exp Incidence/
5. burden.mp. or exp "Global Burden of Disease"/
6. morbidity/ or exp incidence/ or exp prevalence/
7. mortality.mp. or exp "cause of death"/ or exp fatal outcome/ or exp hospital mortality/ or exp survival rate/
8. 2 OR 3 OR 4 OR 5 OR 6 OR 7
9. care home.mp. or exp Home Care Services/
10. nursing home.mp. or exp Respite Care/
11. long term home.mp.
12. residential home.mp. or exp Homes for the Aged/
13. residential care.mp. or exp Homes for the Aged/
14. retirement home.mp. or exp Nursing Homes/ or exp Homes for the Aged/
15. 9 OR 10 OR 11 OR 12 OR 13 OR 14
16. ep.fs.
17. 8 OR 15 OR 16
18. 1 AND 17
19. limit 18 to (humans and yr="2000 -2022")

**EMBASE:**

1. respiratory syncytial virus.mp. or exp Human respiratory syncytial virus/or exp Respiratory syncytial pneumovirus/
2. exp respiratory syncytial virus infection/ or exp infection risk/ or exp infection fatality ratio/ or exp respiratory tract infection/ or exp infection control/ or exp infection fatality rate/ or *infection/ or exp infection rate/
3. outbreak.mp. or exp epidemic/
4. exp cumulative incidence/ or incidence.mp.
5. burden.mp. or exp disease burden/
6. morbidity.mp. or exp morbidity/
7. *mortality/ or exp mortality risk/ or exp mortality rate/ or exp excess mortality/
8. 2 OR 3 OR 4 OR 5 OR 6 OR 7
9. care home.mp.
10. nursing home.mp.
11. long term care.mp.
12. residential home.mp.
13. residential care.mp.
14. retirement home.mp. or exp home for the aged/
15. elderly care.mp.
16. 9 OR 10 OR 11 OR 12 OR 13 OR 14 OR 15
17. ep.fs.
18. 8 OR 16 OR 17
19. 1 AND 18
20. limit 19 to (humans and yr="2000 -2022")

**Global Health (1973 onwards):**

1. respiratory syncytial.mp. or Human respiratory syncytial virus.od.
2. infection.mp. or exp infection/
3. outbreak.mp. or exp outbreaks/
4. incidence.mp. or exp disease incidence/ or exp incidence/
5. burden.mp. or respiratory diseases.sh. or exposure.sh. or disease incidence.sh. or disease prevalence.sh. or human diseases.sh.
6. morbidity.mp. or exp morbidity/
7. mortality.mp. or exp mortality/
8. 2 OR 3 OR 4 OR 5 OR 6 OR 7
9. nursing home.mp. or exp nursing homes/
10. care home.mp. or elderly patients.sh. or elderly.sh.
11. long term care.mp. or exp long term care/
12. residential home.mp. or exp residential institutions/
13. home care.sh. or residential care.mp. or elderly.sh.
14. retirement home.mp. or exp retirement homes/
15. elderly home.mp.
16. 9 OR 10 OR 11 OR 12 OR 13 OR 14 OR 15
17. 8 OR 16
18. 1 AND 17
19. Limit 18 to yr="2000 -2022"


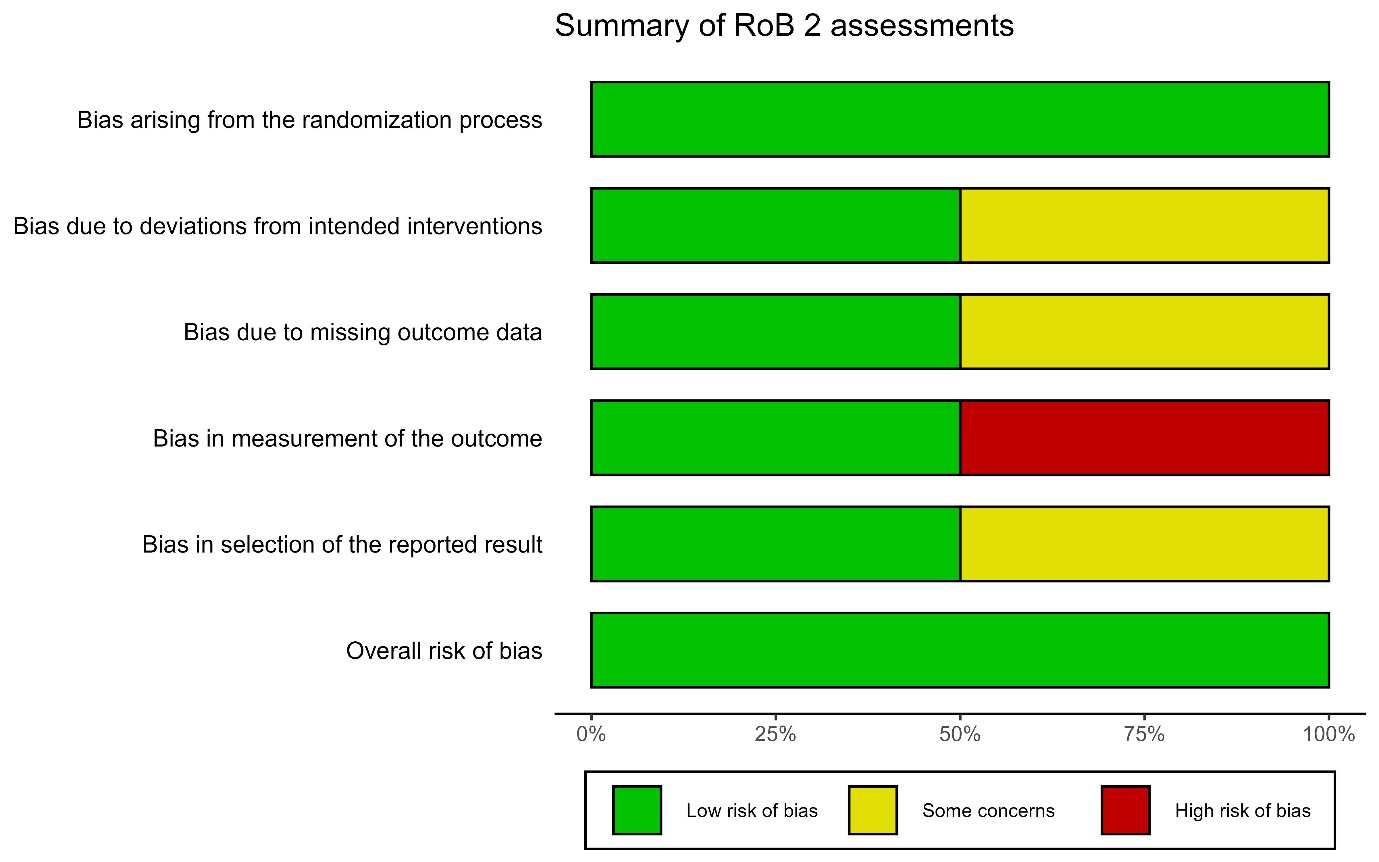


Supplementary Figure 1: Risk of bias assessments of randomised controlled trials using the Cochrane Risk of Bias 2 (RoB 2) tool.

Supplementary Table 1: Risk of bias in non-randomised studies of exposure (ROBINS-E) assessment for observational studies

| Study | D1 | D2 | D3 | D4 | D5 | D6 | D7 | Overall risk |
| --- | --- | --- | --- | --- | --- | --- | --- | --- |
| Barret et al., 2020 | Low | Some concerns | Low | Some concerns | Some concerns | Low | Low | Some concerns |
| Beran et al., 2021 | Low | Low | Low | Low | Low | Some concerns | Low | Low |
| Caram et al., 2009 | Some concerns | Low | Low | Some concerns | Low | Some concerns | Low | Some concerns |
| Chasqueira et al., 2018 | Low | Low | Low | Low | Low | Low | Low | Low |
| Diaz-Decaro et al., 2018 | Some concerns | Low | Low | Low | Some concerns | Low | Low | Low |
| Doi et al., 2014 | Low | Low | Low | No information | Low | Some concerns | Low | Low |
| Ellis et al., 2003 | Low | Some concerns | Low | Some concerns | Some concerns | Some concerns | Some concerns | High |
| Falsey et al., 2008 | Low | Low | Low | Low | Low | Some concerns | Low | Low |
| Hui et al., 2008 | Low | Some concerns | Low | Low | Low | Some concerns | Low | Some concerns |
| Johnstone et al., 2014 | Low | Some concerns | Low | Some concerns | Some concerns | Low | Some concerns | High |
| Loeb et al., 2000 | Low | Some concerns | Low | Low | Low | Some concerns | Low | Low |
| Masse et al., 2017 | Low | Low | Low | Low | Low | Low | Low | Low |
| Meijer et al., 2013 | Some concerns | Low | Low | Some concerns | Low | Low | Low | Low |
| Najeros Pérez et al., 2023 | Low | Low | Low | Low | Low | Low | Low | Low |
| O’Neil et al., 2019 | Some concerns | Low | Low | Low | Low | Low | Low | Low |
| Spires et al., 2017 | Low | Low | Low | Low | Low | Low | Low | Low |
| Ursic et al., 2016 | Low | Low | Low | No information | Some concerns | Low | Low | Low |
| Yip et al., 2018 | Some concerns | Low | Low | Low | Low | Some concerns | Some concerns | Some concerns |

**Domains: Judgement:**

D1: Risk of bias due to confounding Very High risk

D2: Risk of bias arising from measurement of exposure High risk

D3: Risk of bias in selecting participants into the study Some concerns

D4: Risk of bias due to post-exposure interventions Low risk of bias except for concerns about uncontrolled confounding

D5: Risk of bias due to missing data No information

D6: Risk of bias arising from measurement of the outcome

D7: Risk of bias in selection of the reported result
